# Detection of minority drug resistant mutations in Malawian HIV-1 subtype C-positive patients initiating and on first-line antiretroviral therapy

**DOI:** 10.4102/ajlm.v7i1.708

**Published:** 2018-05-30

**Authors:** Zhiyong Zhou, Kevin Tang, Guoqing Zhang, Nellie Wadonda-Kabondo, Kundai Moyo, Lori A. Rowe, Joshua R. DeVos, Nick Wagar, Du-Ping Zheng, Hongxiong Guo, John Nkengasong, Mike Frace, Scott Sammons, Chunfu Yang

**Affiliations:** 1 International Laboratory Branch, Division of Global HIV & TB, Center for Global Health, Centers for Disease Control and Prevention, Atlanta, Georgia, United States; 2 Biotechnology Core Facility Branch, Division of Scientific Resources, Centers for Disease Control and Prevention, Atlanta, Georgia, United States; 3 Department of Preventive Health, Ministry of Health, Lilongwe, Malawi

## Abstract

**Background:**

Minority drug resistance mutations (DRMs) that are often missed by Sanger sequencing are clinically significant, as they can cause virologic failure in individuals treated with antiretroviral therapy (ART) drugs.

**Objective:**

This study aimed to estimate the prevalence of minor DRMs among patients enrolled in a Malawi HIV drug resistance monitoring survey at baseline and at one year after initiation of ART.

**Methods:**

Forty-one plasma specimens collected from HIV-1 subtype C-positive patients and seven clonal control samples were analysed using ultra-deep sequencing technology.

**Results:**

Deep sequencing identified all 72 DRMs detected by Sanger sequencing at the level of ≥20% and 79 additional minority DRMs at the level of < 20% from the 41 Malawian clinical specimens. Overall, DRMs were detected in 85% of pre-ART and 90.5% of virologic failure patients by deep sequencing. Among pre-ART patients, deep sequencing identified a statistically significant higher prevalence of DRMs to nucleoside reverse transcriptase inhibitors (NRTIs) compared with Sanger sequencing. The difference was mainly due to the high prevalence of minority K65R and M184I mutations. Most virologic failure patients harboured DRMs against both NRTIs and non-nucleoside reverse transcriptase inhibitors (NNRTIs). These minority DRMs contributed to the increased or enhanced virologic failures in these patients.

**Conclusion:**

The results revealed the presence of minority DRMs to NRTIs and NNRTIs in specimens collected at baseline and virologic failure time points. These minority DRMs not only increased resistance levels to NRTIs and NNRTIs for the prescribed ART, but also expanded resistance to additional major first-line ART drugs. This study suggested that drug resistance testing that uses more sensitive technologies, is needed in this setting.

## Introduction

Rapid scale-up of antiretroviral therapy (ART) over the past decade has remarkably reduced the mortality and morbidity of HIV-positive patients and decreased HIV transmission. Seventeen million HIV-1-positive patients around the world were receiving ART by the end of 2015.^1^ However, the scale-up of ART in resource-limited settings without adequate treatment monitoring has raised concern about the development of HIV drug resistance. The quasi-species nature of HIV-1 makes the detection of drug resistant mutations (DRMs) more difficult, because the commonly-used Sanger sequencing for drug resistance testing is incapable of detecting these drug resistant HIV variants at a level of less than 20% of the viral population.^2,3,4,5^

Minority drug resistant variants (also known as low-frequency mutants) that are not detected by Sanger sequencing are clinically important, as they can cause virologic failure in patients treated with ART for the first time.^6,7,8,9^ Recent studies have demonstrated that particular drug resistant HIV mutant viruses are clinically significant at a level of 1% of the viral population, as the minority variants can replicate quickly and become the predominant viral population through the selective pressure of ART drugs, leading to treatment failure.^9,10^ However, in the absence of drug pressure in treatment-naïve patients, the stability of transmitted DRMs is different.^11^ For instance, a transmitted M184V mutation can quickly revert to wild-type due to diminished viral fitness.^12^ In patients on ART, minority DRMs may persist for months or years during and post-ART.^13,14,15^ These minority DRMs, not detected by Sanger sequencing, present a need for more sensitive methods to detect the minority DRMs in a clinical sample.

Deep sequencing or next-generation sequencing technologies are extensively used to examine HIV viral diversity and minority drug resistant variants. Next-generation sequencing is a highly sensitive and high-throughput sequencing platform. It can detect HIV variants that make up 0.05% to 1% of viral populations.^16,17,18,19,20,21^

As part of HIV drug resistance surveillance by the Malawi Ministry of Health, a prospective cohort study to monitor ART outcomes and drug resistance development was conducted among patients from ART initiation to one year later. In this 2008 ART patient monitoring survey, 6.1% of the patients on ART for 12–15 months harboured drug resistant HIV.^22^ The most common non-nucleoside reverse transcriptase inhibitor (NNRTI) mutations were K103N (58.1%), Y181C (41.9%) and G190A (6.5%), and the most frequent nucleoside reverse transcriptase inhibitor (NRTI) mutation was M184V (61.3%). The DRMs conferring resistance against NNRTI at baseline were associated with DRMs detected at 12–15 months on ART.^22^ The present study aimed to evaluate parallel tagged deep sequencing primers on clinical samples and to investigate the profile of minority DRMs and their association with virologic failure in the same Malawi ART monitoring cohort.

## Methods

### Ethical considerations

The study protocol was approved by the National Health Sciences Research Committee of Malawi Institutional Review Board (#1001). The use of de-identified data and drug resistance testing using Sanger sequencing and Roche 454 deep sequencing at the Centers for Disease Control and Prevention’s (CDC) global HIV drug resistance laboratory, was determined to be non-human subjects research under CDC protocol #6501 by the Office of the Associate Director for Science at the Center for Global Health, CDC, Atlanta, Georgia, United States.

### Clinical samples

Between February and June 2008, HIV-1-positive patients aged 15 years or older, who initiated first-line ART at four ART clinics following the Malawi ART guidelines, were enrolled. Patients were treated with a first-line regimen combination of stavudine, lamivudine and nevirapine, or an alternative first-line regimen of stavudine to zidovudine substitutions in case of toxicity. Plasma specimens were collected before ART initiation and at 12–15 months on ART for viral load and HIV drug resistance testing.^22^ In the present study, we selected plasma specimens that had enough volume available to evaluate the assay. These were 20 samples collected from participants before ART initiation with viral loads ranging from 10 471 to 2 041 738 copies/mL and 21 samples collected from ART patients at virologic failure (defined as viral load ≥1000 copies/mL) after 12–15 months on ART (viral load ranging from 1738 to 776 247 copies/mL). In addition, six plasmid clones and one mixed clone containing 1% mutant (2495 copies/µL) under the background of a wild-type clone were prepared and used to verify sequence accuracy in this study. All of these plasmid clones were derived from the Malawian cohort samples. All plasmids were constructed using TOPO^TM^ vectors in *Escherichia coli* (Thermo Fisher Scientific, Carlsbad, California, United States). The six wild-type plasmid clones contained the HIV-1 *pol* gene without any DRMs, and the mutant clone contained DRMs at codons 103, 181, 184 and 190 of the HIV-1 *pol* gene.

### Viral ribonucleic acid extraction and reverse transcriptase polymerase chain reaction

RNA was extracted from plasma specimens using the automated Abbott™ Sample Preparation System (m2000sp) (Abbott Laboratories. Abbott Park, Illinois, United States). The copy number of HIV-1 RNA was measured using RT-qPCR on the m2000rt Real Time Analyzer (Abbott Laboratories. Abbott Park, Illinois, United States).^22^ The viral RNA was subjected to one-step RT-PCR amplification as described previously.^11^

### Parallel deep sequencing

Degenerate primers, capable of amplifying multiple HIV-1 group M subtypes, were designed based on the HIV-1 *pol* gene sequences (www.hiv.lanl.gov) (Table 1). Six overlapping primer sets (forward and reverse) were used for bidirectional coverage of protease amino acids 6 to 99 and reverse transcriptase amino acids 1 to 251. The size of the assembled gene fragment was 1035 base pairs. These six primers, tailed with Roche 454 adaptor and multiplex identifier sequences (tags), were synthesised at the CDC Biotechnology Core Facility. For PCR amplification, a 50µL reaction contained 1× AccuPrime PCR Buffer II (Thermo Fisher Scientific, Carlsbad, California, United States), 0.5 U AccuPrime Taq High Fidelity (Thermo Fisher Scientific, Carlsbad, California, United States), 13.5 µL water, 0.3 µM, each forward and reverse primer, and 2 µL DNA. All reactions were performed in 9700 thermal cyclers (Applied Biosystems, Austin, Texas, United States) under the following program: 95°C for 10 minutes (min); five cycles of 94°C for 20 seconds (s), 48°C for 20 s, and 72°C for 1 min for annealing the primers with unique molecular tags followed by 35 cycles of 94°C for 30 s, 60°C for 20 s, 72°C for 30 s; and one cycle of 72°C for 5 min. The reaction products were confirmed by 1% agarose gel electrophoresis. The amplicons were purified using the Agencourt AMPure XP beads (Beckman Coulter, Beverly, Massachusetts, United States) or QIAGEN gel purification kits (QIAGEN, Germantown, Maryland, United States) and then quantified using the Quant-iT PicoGreen dsDNA kit (Thermo Fisher Scientific, Carlsbad, California, United States). Each sample had its own unique tag sequences for its six amplicons. Six barcoded samples were pooled and sequenced in one region. A total of eight regions were used for 48 samples on a plate. PCR amplicons from six samples were pooled in equal amounts, and amplified in water-in-oil emulsion PCR at the CDC Biotechnology Core Facility. Amplicons of 41 field samples and seven plasmid DNA samples were sequenced on the 454 platform (GS-FLX, Roche Applied Science, Indianapolis, Indiana, United States).

**TABLE 1:** HIV-1 primers designed and used for amplifying *pol* gene amplicons with the deep sequencing method.

Amplicon name	Primer name	Primer sequence	HXB2 position
Amplicon 1	PR1F	CTTTARCTTCCCTCARATCACTCT	2243–2266
	PR1R	TCTTCCAATTATGTTGACAGG	2513–2493
Amplicon 2	PR2F	ATGGAAACCAARAATGATAG	2375–2394
	PR2R	TTYTCTTCTGTYAATGGCCA	2638–2619
Amplicon 3	RT1F	AGTCCTATTGARACTGTRCCAGT	2556–2578
	RT1R	CTGAAATCTACTAATTTYCTCCA	2782–2760
Amplicon 4	RT2F	AATTGGGCCTGAAAATCCATAYAAIACTCC	2696–2725
	RT2R	GGAATATTGCIGGTGATCCTTTCC	3030–3007
Amplicon 5	RT3F	ACAGTACTRGATGTGGGKGATGCATA	2868–2893
	RT3R	TATTTCTAARTCAGATCCTACATA	3134–3111
Amplicon 6	RT4F	CAATATTCCARAGTAGCATGAC	3022–3043
	RT4R	TTCTGTATRTCATTGACAGTCCA	3325–3303

### Deep sequencing analysis

Sequence files generated by Roche 454 deep sequencing were analysed using GS Amplicon Variant Analyzer pipeline from Roche Applied Science (Indianapolis, Indiana, United States). The deep sequencing analysis process in the present study included quality restriction for base call setting at 60 for signal intensity. All amplicon reads of alignment and single nucleotide polymorphism (SNP), calling against the HXB2 reference sequence, were evaluated with a quality score ≥ 25 and read length ≥ 220 base pairs. Sequence accuracy of Roche 454 runs was evaluated using the sequences generated by Sanger sequencing of the seven plasmid clones. The minority variant was defined as a SNP detected at > 0.68% and < 20% of the frequency of mutations. For DRM analysis, mutations were called and grouped based on International AIDS Society (IAS)-USA 2011 recommendations.^23^ Additional mutations for those samples collected before ART initiation were analysed based on the 2009 World Health Organization surveillance DRM list.^24^

### Standard Sanger sequencing

The standard genotyping of HIV drug resistance was performed on all plasma samples using an in-house population-based sequencing assay.^11^ Raw sequencing data were analysed using the customised ReCall software, v.2.24 (provided by Dr. Richard Harrigan from British Columbia’s Center for Excellence in HIV/AIDS Research, Vancouver, Canada).^25^ The mixed mutation calling threshold was set at ≥ 20% of the main peak. The DRMs were analysed as stated above for parallel deep sequencing results.

### Statistical analysis

All statistical analysis was performed using SPSS Statistics v19 (SPSS Inc., Chicago, Illinois, United States). The Wilcoxon Signed-Rank test was used to analyse the statistical differences in the number of DRMs detected between Sanger sequencing and deep sequencing. Fisher’s exact test was used to compare the prevalence of HIV drug resistance by these two methods. A *P*-value of < 0.05 was considered statistically significant.

## Results

### Parallel deep sequencing coverage and estimate of sequence errors

**FIGURE 1:**
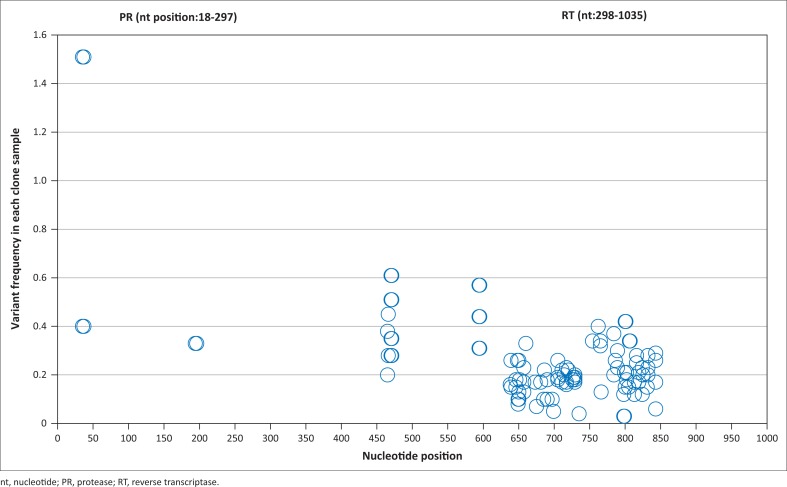
Error frequency of 454 deep sequencing on the six plasmid control clones at each nucleotide position from amplified amplicons. The X axis is the nucleotide sequence location in protease and reverse transcriptase genes; the Y axis is the percentage of single nucleotide polymorphism error.

The GS-FLX deep sequencing of a single run yielded 246 849 raw sequence reads, of which 242 246 (98.1%) reads passed the quality restrictions with a mean read length of 270 nucleotides, while 4603 (1.9%) low-quality sequences were removed from the analysis pipeline. On average, 5490 reads per sample were obtained (range from 420 to 5673 reads). From the 1% mixed clone, K103N was not detected within the 520 sequence reads; Y181C and M184V were detected at the level of 1.03% with 1335 reads, while G190A was detected at 0.97% with 785 reads. The mean error rate plus two standard deviations was 0.43 from the six control plasmid clones. Sequence errors mostly occurred at the overlapping areas of amplicons in the *RT* gene. There was only one SNP showing an error rate of 0.68% at codon 17 of the *PR* gene in a polyG region (GGGG**G**GCA, nucleotide 35, Figure 1). This error at *PR* codon 17 was not in the DRM position according to IAS and Stanford HIV database definitions. Another high-error site was codons 63 of the *RT* gene (nucleotides 470 and 471, Figure 1), with a 0.61% error rate. Based on these background errors for each nucleotide position, the frequency > 0.68% error rate was used as the threshold for true SNPs when evaluating the minority DRMs in the clinical samples.

### Comparative analysis of drug resistance mutations detected by parallel deep sequencing and Sanger sequencing

**FIGURE 2:**
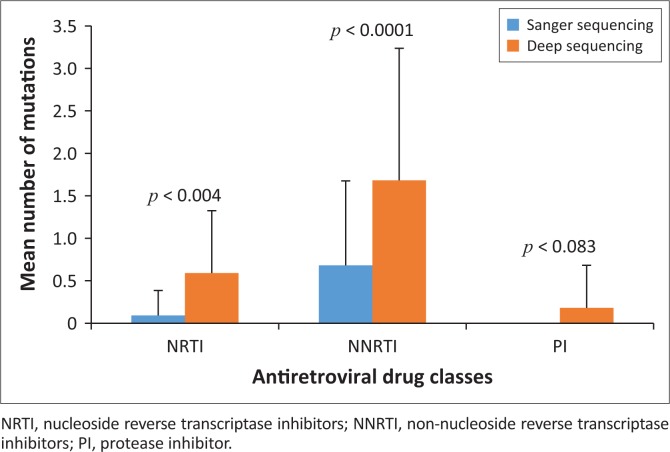
Comparison of mean number of HIV-1 drug resistant mutations detected by Sanger and deep sequencing methods. The vertical bars present mean number of mutations ± standard error; *p* < 0.05 was considered statistically significant.

Barcoded deep sequencing primers amplified all amplicons from the clinical samples. Roche GS-FLX deep sequencing identified all 72 of the DRMs that had been detected by Sanger sequencing at a level of ≥ 20% from the 41 clinical Malawian samples. Additionally, a total of 79 DRMs were exclusively detected by deep sequencing. Thus, Sanger sequencing missed 52.3% of DRMs at a level of < 20% in these clinical samples. The differences in the numbers of DRMs detected by these two sequencing approaches were significant against NRTIs (*p* = 0.004) and NNRTIs (*p* = 0.0001). Further, the Cohen’s effect size value (*d* = 0.91 and 0.79) suggests a moderate to high practical significance. No significant difference was found in detection of protease inhibitor (PI) mutations (*p* = 0.083) (Figure 2).

### Prevalence of minority drug resistance mutations in baseline samples before antiretroviral therapy initiation

Overall, the frequency of DRMs increased by deep sequencing with minority DRMs being detected in 17 (85%) of the 20 pre-ART samples by deep sequencing. Minority NRTI were detected in 10 (50%) of these samples and NNRTI mutations in 14 (70%), while minority PI mutations were found in three (15%) of the 20 samples. In addition, the number of DRMs detected by deep sequencing was higher than Sanger sequencing. This increase of DRM numbers detected by deep sequencing was statistically significant for NRTI (2/20 vs 13/20, *p* = 0.015), but not for NNRTI (9/20 vs 14/20, *p* = 0.13) or PI (0/20 vs 3/20, *p* = 0.235) when compared to Sanger sequencing. The most common minority NRTI mutation was K65R (6 of 20), followed by M184I (2 of 20, Table 2). The SNP frequency for K65R ranged from 0.7% to 2.5% of the sequence reads in these six patients and both mutant alleles of AGG and AGA were detected in these HIV-1 subtype C-positive samples. The common minority NNRTI mutations detected were V106M, V179T and G190A (3/20 each), followed by E138K and Y181C (2/20 each), and K101P, K103N, V108I, E138A, V179D, A190E and H221Y (1/20 each). The minority PI mutations detected in three of the samples were M46I (2/20) and D30N (1/20) (Table 2).

**TABLE 2:** Number of drug resistance mutations detected by two sequencing methods against PIs, NRTIs and NNRTIs from plasma specimens collected from patients before ART initiation and at virologic failure†

Drug class	Mutation	Before ART initiation (*n* = 20)			At virologic failure (*n* = 21)		
		No. of mutations detected by Sanger	No. of mutations detected by NGS	No. of mutations detected by NGS only	No. of mutations detected by Sanger	No. of mutations detected by NGS	No. of mutations detected by NGS only
PI	D30N	0	1	1	0	0	0
	M46I	0	2	2	0	2	2
	M46L	0	0	0	0	1	1
**Subtotal**		**0**	**3**	**3**	**0**	**3**	**3**
NRTI	M41L	0	0	0	0	1	1
	E44D	1	1	0	1	1	0
	A62V	0	0	0	2	3	1
	K65R	0	6	6	2	4	2
	D67N	0	0	0	3	4	1
	T69D	0	0	0	0	1	1
	K70R	1	1	0	0	0	0
	V75M	0	0	0	0	1	1
	V118I	1	1	0	2	2	0
	M184I	0	2	2	0	3	3
	M184V	0	0	0	11	13	2
	T215F	0	0	0	0	1	1
	K219E	0	0	0	0	1	1
**Subtotal**		**3**	**11**	**8**	**21**	**35**	**14**
NNRTI	V90I	1	1	0	0	2	2
	A98G	0	0	0	1	1	0
	K101E	2	2	0	1	4	3
	K101P	0	1	1	0	0	0
	K103N	2	3	1	9	11	2
	V106A	0	0	0	0	1	1
	V106M	0	3	3	1	2	1
	V108I	0	1	1	3	7	4
	E138A	2	3	1	2	4	2
	E138K	0	2	2	0	0	0
	V179D	0	1	1	1	1	0
	V179E	0	0	0	0	1	1
	V179T	1	4	3	0	4	4
	Y181C	5	7	2	9	10	1
	Y188C	0	0	0	0	2	2
	Y188L	0	0	0	0	1	1
	G190A	1	4	3	1	4	3
	G190E	0	1	1	0	1	1
	G190R	0	0	0	1	1	0
	H221Y	1	2	1	4	7	3
**Subtotal**		**15**	**35**	**20**	**33**	**64**	**31**

ART, antiretroviral therapy; NGS, next-generation sequencing; PI, protease inhibitor; NRTI, nucleoside reverse transcriptase inhibitor; NNRTI, non-nucleoside reverse transcriptase inhibitor.

† Virologic failure was defined as plasma viral load ≥1000 copies/mL after 12–15 months on ART.

### Prevalence of minority drug resistance mutations in patients experiencing virologic failure after antiretroviral therapy

Overall, minority DRMs were detected by deep sequencing in 90.5% (19/21) of the patients failing ART. Of the 21 Malawian samples, minority NRTI mutations were detected in 8 (38%) samples, while minority NNRTI mutations were detected in 14 (66.7%) samples (Table 2). Minority PI mutations were only detected in 3 of 21 (14.3%) samples. However, the increased number of minority mutations in individual patients for NRTI or NNRTI in ART-failing patients was not statistically significant (NRTI: 12/21 vs 14/21, *p* > 0.05; or NNRTI: 18/21 vs 19/21, *p* > 0.05) when compared to Sanger sequencing. Among the minority NRTI mutations, M184I was detected in three samples, and M184V and K65R in two samples respectively. Other minority mutations including M41L, A62V, D67N, T69D, V75M, T215F and K219E were found in one sample each. Among the minority NNRTI mutations, V108I, V179T were detected in four samples and G190A, H221Y and K101E were detected in three samples. Other NNRTI mutations, such as V90I, K103N, V106A/M, E138A, V179E, Y181C, Y188C/L and G190E were also found in one or two samples.

### Clinical impact of minority drug resistance mutations detected by deep sequencing on virologic failure

To study the impact of minority DRMs detected by deep sequencing on the clinical outcome of patients on ART, we compared drug resistance levels (genotype susceptibility score) or expansion of drug resistance to additional drugs or drug classes from those 21 ART-failure patients against NRTIs and NNRTIs using the Stanford HIV drug resistance database tool. Among 19 patients with the additional minority DRMs detected, we found that seven (36.8%) of the patients had enhanced resistance levels to NRTIs. More importantly, three of the seven patients had gained low- to high-level resistance against tenofovir (Table 3), a key component of the current World Health Organization-recommended first- and second-line regimens.^26^ Fourteen (73.7%) out of the 19 patients also had an enhanced resistance level to NNRTIs and 13 of these 14 patients had intensified or expanded resistance profiles against the second generation of NNRTIs (etravirine and rilpivirine) (Table 4). Furthermore, the drug resistance profile analyses revealed an intermediate- or high-level resistance to the relevant first-line regimens (stavudine, lamivudine and nevirapine or zidovudine, lamivudine and nevirapine) that are prescribed to these Malawian patients and which might explain the virologic failures these patients experienced.

**TABLE 3:** Comparison of NRTI drug resistance mutations detected from plasma specimens collected from patients with virologic failure after 12–15 months on ART by Sanger sequencing and deep sequencing and their impacts on NRTI susceptibility.

Sample ID	Mutations by Sanger	Drug resistance level (score)†	Mutations by deep sequencing	Drug resistance level (score)
5426	D67N, M184V	3TC(60),ABC(15),DDI(15),FTC(60)	K65R,D67N, M184V	*3TC(90),ABC(65),D4T(60),DDI(75), FTC(90),TDF(65)*
5471	A62V,K65R,D67N,M184V	3TC(95),ABC(70),D4T(65),DDI(80), FTC(95),TDF(80)	M41L,K219E,A62V,K65R,D67N,M184V	*3TC(100),ABC(85),D4T(90),DDI(95),* *FTC(100),TDF(95),AZT(20)*
5496	A62V,V75I,M184V	3TC(70),ABC(25),DDI(20),FTC(70)	A62V,V75I,M184V,T69D,T215F	*3TC(75),ABC(40),AZT(45),D4T(55),DDI(65),FTC(75),TDF(15)*
5506	M184V	3TC(60),ABC(15),DDI(10),FTC(60)	M184V	3TC(60),ABC(15),DDI(10),FTC(60)
5549	M184V	3TC(60),ABC(15),DDI(10),FTC(60)	D67N,M184V	3TC(60),*ABC(20),DDI(15)*,FTC(60)
5642	M184V	3TC(60),ABC(15),DDI(10),FTC(60)	M184I,M184V	3TC(60),ABC(15),DDI(10),FTC(60)
5727	M184V	3TC(60),ABC(15),DDI(10),FTC(60)	K65R,D67N,V75M,M184I,M184V	*3TC(90),ABC(65),D4T(100),DDI(90),FTC(90),TDF(65)*
6525	E44D,T69N,M184V	3TC(60),ABC(15),DDI(20),FTC(60)	E44D,T69N,M184V	3TC(60),ABC(15),DDI(20),FTC(60)
5713	V118I, M184V	3TC(60),ABC(15),DDI(10),FTC(60)	A62V,V118I,M184V	*3TC(65),ABC(20),DDI(15),FTC(65)*
5597	None	S	M184V	*3TC(60),ABC(15),DDI(10),FTC(60)*
5527	M184V	3TC(60),ABC(15),DDI(10),FTC(60)	M184V	3TC(60),ABC(15),DDI(10),FTC(60)
5508	K65R, D67N	3TC(30),ABC(50),D4T(60),DDI(65), FTC(30),TDF(65)	K65R,D67N	3TC(30),ABC(50),D4T(60),DDI(65),FTC(30),TDF(65)
5715	M184V	3TC(60),ABC(15),DDI(10),FTC(60)	M184V	3TC(60),ABC(15),DDI(10),FTC(60)
5434	None	S	M184I,M184V	*3TC(60),ABC(15),DDI(10),FTC(60)*

ABC, abacavir; AZT, zidovudine; DDI, didanosine; FTC, emtricitabine; 3TC, lamivudine; TDF, tenofovir; d4T, stavudine.

† , Scores 10–14: potential low-level of resistance; 15–29, low-level of resistance; 30–59, intermediate-level of resistance; ≥60, high-level of resistance;

S, susceptible (0–9). The letters and numbers in italics indicate increased/expanded levels of drug resistance caused by minority drug resistance mutations detected by deep sequencing only

## Discussion

The present study has shown that massively parallel deep sequencing is capable of detecting minority HIV-1 variants from HIV-1 subtype C clinical samples. The higher prevalence of minority DRMs in pre-ART Malawian patients to NRTIs by deep sequencing was statistically significant compared to Sanger sequencing. The minority mutation profile revealed that the increased minority DRMs were associated with enhanced DR levels in virologically failing patients.

This study was designed to evaluate parallel tagged deep sequencing in detection of minority DRMs in samples collected from Malawi. We successfully amplified all 41 plasma specimens collected from the patients and seven plasmid DNA samples that generated 242 246 sequencing reads using the degenerate primers designed for HIV-1 group M subtypes and circulating recombinant forms. Our results not only showed 100% concordance of DRMs detected by Sanger sequencing and deep sequencing, but deep sequencing also detected over 50% minority DRMs in these clinical samples. At the lower detection level of 0.68%, set by the current study, minority DRMs were detected in a majority of samples collected from patients before ART initiation and at virologic failure. Although we only analysed HIV-1 subtype C-positive Malawian samples in this study, the primers were designed for all relevant HIV-1 group M subtypes and circulating recombinant forms. In fact, we were able to amplify subtype B, B/C, F and G samples using these primers which were confirmed by gel electrophoresis and successfully detect 5 of 5 subtype B samples using deep sequencing methods.^27^

Previous studies reported the use of parallel tagged deep sequencing methods in detecting low levels of HIV-1 subtype B variants.^18,20,21,28,29^ A study by Dudley et al.^20^ evaluated a 454 GS Junior sequencer by multiplexing 48 samples collected from HIV-1 subtype B-positive individuals and obtained a sequencing success rate of 93% and an error rate of 0.71%. Our study using GS-FLX with multiplexing on 48 samples collected from HIV-1 subtype C-positive patients showed a 100% amplification rate and 0.265% mean error rate. Many studies have reported that the mean error rate of pyrosequencing techniques can be down to 0.05% to 1%.^17,19,21,30^ The error rates for deep sequencing not only affect the accuracy of base calling, but also impact the sensitivity of minority variant detection. It has been reported that factors, such as the input number of template molecules, sequence primers, amplicon length, nucleotide sequences, PCR errors and operational procedures, might contribute to deep sequencing assay sensitivity and accuracy.^17,31,32,33^ In addition, error rates are nucleotide position-dependent and Roche 454 deep sequencing methods are prone to have more errors at the homopolymeric regions.^17,31^ In the present study, cross-over errors of major DRMs between samples were not found. To balance the detection sensitivity with detection accuracy, we set up the base calling threshold for low-frequency mutations at > 0.68% (mean error rate + 2 standard deviations) which was calculated based on the error rates of individual nucleotide positions of six control plasmid samples. In the present study, the K103N mutation was not detected from the mixed clone at 1% of minority variant level of control plasmids from 520 reads. This was likely due to not having enough reads amplified for this mixed plasmid as a previous study demonstrated that at least 1950 reads are required for detecting a minority variant for K103N mutations at about the 1% level.^17^ One limitation of our study was that the primer pair for amplifying amplicons containing codon 103 of *RT* gene was not optimal for the depth of coverage. Some minority K103N mutations could have been missed in this study. Thus, the number of sequence reads obtained for each nucleotide position and errors at the homopolymeric regions played an important role in the depth of the next-generation sequencing.

Our results from samples collected from patients failing ART and initiating ART demonstrate that deep sequencing has the added benefit of detecting low-frequency mutations in this Malawian cohort. Overall, deep sequencing detected significantly more DRMs than Sanger sequencing. Of those specimens collected from patients initiating ART, more DRMs against NRTI were detected by deep sequencing. Among the minority DRMs, detected by 454 deep sequencing, K65R and M184I were the most common and may compromise the effectiveness of both first- and second-line drugs used according to the Malawi ART guidelines. The K65R mutation can confer resistance to stavudine and cross-resistance to lamivudine, abacavir, emtricitabine and tenofovir,^23,34,35^ and is more frequently identified in HIV-1 subtype C.^30,35,36^ Similar to previous studies, the K65R mutation was seen in both treatment-naïve patients and patients failing ART in this cohort. Several studies have reported that increased presence of K65R mutations is caused by pyrosequencing errors or by the nucleotide template of subtype C viruses (such as the ATA sequence at codon 63 of the *RT* gene).^30,32,37^ Even though no errors at codon 65 of the *RT* gene were found by deep sequencing in the current study, we did find a relatively higher error rate at codon 63 of the *RT* gene in the subtype C plasmid sequences. However, we did not find higher error rates for K65R compared with other DRM sites in these patients. The M184I mutations were only detected at low frequencies by deep sequencing in pre-ART patients and patients with treatment failure. M184I was considered to be a transient mutation before being replaced by M184V.^19,38,39^ No detectable levels of M184V mutations were found in pre-ART samples using deep sequencing, but M184V mutation was detected in over three-quarters of samples from patients failing ART. Taken together, the higher NRTI resistance mutations of M184V and K65R in patients failing ART were more likely acquired by selective drug pressure in this Malawian cohort treated with a regimen containing stavudine and lamivudine.^22^

In this study, most samples collected from virologic failure patients had detectable DRMs to NNRTI by both Sanger sequencing and deep sequencing. The mutations K101E, K103N, V106A/M, V179D/T, Y181C, G190A/E and H221Y to NNRTI were the most common minority mutations detected in these patients. Virus with K101E/Y181C/G190A and other mutations could increase levels of resistance to nevirapine 893-fold.^40^ The H221Y mutation could also impact clinical outcomes as Y181C/H221Y along with the K103N or K101Q mutations could increase resistance levels to nevirapine over 100-fold (K103N) or 3000-fold (K101Q).^41^ The drug resistance profile generated by deep sequencing revealed that these mutations were associated with the first-line regimen (stavudine, lamivudine and nevirapine). The DRMs to NNRTI in pre-ART samples were also relatively high in these patients and were likely due to single-dose nevirapine used in the prevention of mother-to-child transmission program in Malawi.^22^ However, our results could not rule out the presence of transmitted drug resistance to NNRTI in these pre-ART patients.

Although DRMs against PIs were not detected using Sanger sequencing, they were detected by deep sequencing in this cohort. M46I/L is considered a major PI mutation and would increase drug resistance levels to PIs along with other mutations.^34,42^ Because no PI drugs were used in the first-line ART in this cohort, these PI mutations were likely natural polymorphisms of HIV-1. The natural polymorphism of M46I has been reported to have a replicative advantage for subtype B,^43^ while the impact of M46I/L natural polymorphisms on the development of drug resistance in patients is unknown. As Malawi has started to use lopinavir/ritonavir for second-line regimens,^44^ the emergence of DRMs to PIs should be closely monitored.

Evidence is lacking in understanding the real clinical impact of minority DRMs. Clinical trials are needed to accurately evaluate the clinical consequences of these DRMs. However, our results indicate that minority mutations detected by barcoded deep sequencing show an increased or expanded level of resistance to NRTIs and NNRTIs (see mutation scores in Tables 3 and 4). For instance, increased M184IV/I mutations would reduce susceptibility to lamivudine and emtricitabine (scores from 0 to 60, from susceptible to high-level of resistance). K65R+ M184V/I would reduce susceptibility to tenofovir and didanosine from low-level (scores from 0 to 60) to high-level resistance (scores from 15 to 75). Additionally, other individual thymidine-analog mutations showed an intermediate or high level of resistance to Malawi’s first-line regimens (stavudine, lamivudine and nevirapine). These low-frequency mutations detected by the barcoded parallel sequencing added significant values to the resistant reservoir in the HIV-positive population. All mutation data, both majority and minority mutations, can be used by doctors or policy makers as a reference when changing or revising treatment therapy for patients with virologic failure or country first-line regimens in Malawi.

**TABLE 4:** Comparison of NNRTI drug resistance mutations detected from plasma specimens collected from patients with virologic failure after 12–15 months on ART by Sanger sequencing and deep sequencing and their impacts on NNRTI susceptibility.

Sample ID	Mutations by Sanger	Drug resistance level (score)†	Mutations by NGS	Drug resistance level (score)
5426	Y181C,H221Y	EFV(40),ETR(40),NVP(70),RPV(40)	Y181C,H221Y	EFV(40),ETR(40),NVP(70),RPV(40)
5471	Y106M,Y181C	EFV(90),NVP(120),ETR(30),RPV(30)	Y106M,Y181C	EFV(90),NVP(120),ETR(30),RPV(30)
5496	K103N	EFV(60),NVP(60)	V108I,K103N,E138A,H221Y,	*EFV(80),NVP(85),ETR(20),RPV(25)*
5506	K103N,Y181C,G190A,H221Y	EFV(145),ETR(65),NVP(190),RPV(65)	K101E,K103N,V108I,Y181C,G190A,H221Y	*EFV(170),ETR(80),NVP(235),RPV(95)*
5549	Y181C,G190R	EFV(30),ETR(30),NVP(60),RPV(30)	V179E,Y181C,Y188C G190R	*EFV(100),ETR(40),NVP(130),RPV(40)*
5642	K103N	EFV(60),NVP(60)	K103N	EFV(60),NVP(60)
5727	K103N,Y181C,H221Y	EFV(100),ETR(40),NVP(130),RPV(40)	V90I,K103N,V179T,K138T,Y181C,H221Y	*EFV(110),ETR(50),NVP(140),RPV(50)*
6525	K101E,E138A,Y181I	EFV(45),ETR(85),NVP(90),RPV(105)	K101E,E138A,V179T,Y181I,H221Y	*EFV(65),ETR(105),NVP(110),RPV(125)*
5713	Y108I,Y181C,H221Y	EFV(50),ETR(40),NVP(85),RPV(40)	Y108I,V179T,Y181C,H221Y	*EFV(60),ETR(50),NVP(95),RPV(50)*
5585	V179D	EFV(10),ETR(10),NVP(10),RPV(10)	V179D	EFV(10),ETR(10),NVP(10),RPV(10)
5597	K103N	EFV(60),NVP(60)	K103N,V108I,E138A	*EFV(70),NVP(75),RPV(15)*
5778	None	S	G190E	*EFV(60),NVP(60),ETR(45),RPV(45)*
5527	A98G,Y181C	EFV(40),ETR(40),NVP(90),RPV(45)	A98G,K101E,V108I,V179T,Y181C,H221Y	*EFV(85),ETR(75),NVP(155),RPV(95)*
5665	E138A	RPV(15)	K103N,E138A	*EFV(60),NVP(60)*,RPV(15)
5439	Y181C	EFV(30),ETR(30),NVP(60),RPV(30)	K103N,V106A,V106M,Y181C,Y188C,G190A	*EFV(255),ETR(55),NVP(300),RPV(55)*
5608	K103N	EFV(60),NVP(60)	K103N,G190A	*EFV(105),NVP(120),ETR(15),RPV(15)*
5508	K103N,Y108I	EFV(70),NVP(75)	V90I,K103N,Y108I,Y181C,Y188L	*EFV(160),NVP(195),ETR(45),RPV(90)*
5715	K103N,V108I	EFV(70),NVP(75)	K101E,K103N,V108I,G190A	*EFV(130),NVP(165),ETR(30),RPV(35)*
5434	K103N,Y181C	EFV(90),ETR(30),NVP(120)	K103N,Y181C	EFV(90),ETR(30),NVP(120)

ETR, etravirine; EFV, efavirenz; NVP, nevirapine; NGS, next-generation sequencing; RPV, rilpivirine.

† , Score 10–14: potential low-level of resistance; 15–29, low-level of resistance; 30–59, intermediate-level of resistance; ≥60, high-level of resistance; S, susceptible. The letters and numbers in italics indicate increased/expanded levels of drug resistance caused by minority drug resistance mutations detected by deep sequencing only.

### Limitations

This study had its limitations. First, the sample size was small due to the availability of remnant samples and budget constraints. A statistically-appropriate sample size should be used for population-level estimations of DRMs in order to make a meaningful statement. Second, although significantly increased minority DRMs were observed in the samples collected from pre-ART and patients with virologic failure using Roche 454 barcoded deep sequencing, lack of proper plasmid mutant K65R control in the test might have compromised the accuracy of calculating the K65R mutation rate. Third, some methods in the current study could not be applied further as a result of the 454 platform and technologies being discontinued due to high cost and errors at some homopolymeric regions. However, the designed primers and barcoded strategy in the current study could be applied to other deep sequencing platforms for HIV drug resistance testing or studies.

### Conclusion

In conclusion, our study confirmed that barcoded parallel deep sequencing technology is capable of detecting minority DRMs from clinical patient samples. These minority DRMs not only increased resistance levels to the antiretroviral drugs that are being prescribed, but they also expanded resistance to additional major first-line antiretroviral drugs such as tenofovir. The minority DRMs detected by deep sequencing may be helpful for selecting the optimal regimens for patients initiating ART and for patients who fail first-line regimens.
